# PReS-FINAL-2051: The difference of disease's perception by JIA patients and their parents: analysis of the JAMAR questionnaire

**DOI:** 10.1186/1546-0096-11-S2-P64

**Published:** 2013-12-05

**Authors:** F Vanoni, B Fonjallaz, G Lucie, A von Scheven-Gête, M Hofer

**Affiliations:** 1 DMCP-CHUV-Lausanne, Division of pediatric rheumatology, Lausanne, Switzerland

## Introduction

The JAMAR (Juvenile Arthritis Multidimensional Assessment Report) has been developed to evaluate the perception of the patient and his parents on different items: well-being, pain, functional status, quality of life, disease activity, disease course, side effects of medication, therapeutic compliance and satisfaction with illness outcome.

## Objectives

Our aim was to compare disease's perception by JIA patients and their parents.

## Methods

We included 100 consecutive JIA patients-parents pairs; the patients had a median age of 13.1 years and a median age at onset of 7.7 years. The male-female ratio was 1:2.7. The diagnostic distribution of JIA was: 35 enthesitis-related arthritis (35%), 39 oligoarthritis (39%), 14 polyarthritis RF negative (14%), 1 polyarthritis RF positive (1%) 4 systemic (4%). For each patient the JAMAR was filled separately by the child and one parent just before the consultation. We analyzed separately the different components of the JAMAR.

## Results

Table 1 shows the results of data analysis for five items.

**Table 1:** 

Median (range)		VAS P	VAS DA	VAS WB	JAFS	HRQOL
Children Parents		2.5 (0-10) 2 (0-10)	2 (0-10) 2 (0-10)	1.5 (0-10) 2 (0-10)	1(0-13) 1(0-14)	4 (0-24) 4 (0-18)

**Scores (N° of pairs)** Score difference =0 At least one score >0 Score difference >1		50 72 19/72(26%)	47 69 23/69(33%)	46 70 28/70(40%)	59 64 24/64 (38%)	44 84 32/84 (38%)

Child score > Parent score	Diff>0 Diff>1	34 **14**	28 **15**	24 **16**	21 12	33 **14**

Parent score > child score	Diff>0 Diff>1	16 **5**	25 **8**	30 **12**	20 12	23 **18**

VAS P: Visual analogue scale for pain; VAS DA: Visual analogue scale for disease activity; VAS WB: Visual analogue scale for well being; JAFS: juvenile arthritis functional score; HRQOL: health related quality of life.

We evaluated the differences between patients and their parents for each of the 5 different scores. About half of the pairs did not show any difference; among the pairs with at least one score >0, from 26% to 40% of them had a difference >1. For these pairs, children reported more often a higher level of pain, disease activity and well-being than their parents, but the reverse was true for health related quality of life.

## Conclusion

Our observation suggests that the perception of the disease may differ widely depending if the patient or his parents are asked. Further analysis is needed to elucidate if there is a subgroup of patients for whom the differences are more frequent.

## Disclosure of interest

None declared.

